# The relationship between perineal trauma and postpartum psychological outcomes: a secondary analysis of a population-based survey

**DOI:** 10.1186/s12884-023-05950-6

**Published:** 2023-09-06

**Authors:** Charles Opondo, Siân Harrison, Julia Sanders, Maria A. Quigley, Fiona Alderdice

**Affiliations:** 1 NIHR Policy Research Unit in Maternal and Neonatal Health and Care, National Perinatal Epidemiology Unit, Nuffield Department of Population Health, University of Oxford, Old Road Campus, Headington, OX3 7LF Oxford UK; 2 School of Healthcare Sciences, College of Biomedical and Life Sciences, Cardiff University, Ty Dewi Sant Health Park, Cardiff, CF14 4XN UK

**Keywords:** Perineal trauma, Psychological outcomes, Postpartum, Survey

## Abstract

**Background:**

Perineal trauma, involving either naturally occurring tears or episiotomy, is common during childbirth but little is known about its psychological impact. This study aimed to determine the associations between childbirth related perineal trauma and psychological outcomes reported by women three months after giving birth and to explore factors that could mediate relationships between perineal trauma and maternal psychological outcomes.

**Methods:**

This study was a secondary analysis of data from a cross-sectional population-based survey of maternal and infant health. A total of 4,578 women responded to the survey, of which 3,307 had a vaginal birth and were eligible for inclusion into the analysis. Symptoms of depression, anxiety, and post-traumatic stress (PTS) symptoms were assessed using validated self- report measures. Physical symptoms were derived from a checklist and combined to produce a composite physical symptoms score. Regression models were fitted to explore the associations.

**Results:**

Nearly three quarters of women experienced some degree of perineal trauma. Women who experienced perineal trauma reported having more postnatal physical symptoms (adjusted proportional odds ratio 1.47, 95%CI 1.38 to 1.57, *p*-value < 0.001), were more likely to report PTS symptoms (adjusted OR 1.19, 95%CI 1.04 to 1.36, *p*-value 0.010), and there was strong evidence that each unit increase in the physical symptoms score was associated with between 38 and 90% increased adjusted odds of adverse psychological symptoms. There was no evidence of association between perineal trauma and satisfaction with postnatal care, although there was strong evidence that satisfaction with labour and birth was associated with 16% reduced adjusted odds of depression and 30% reduced adjusted odds of PTS symptoms.

**Conclusions:**

Women who experienced perineal trauma were more likely to experience physical symptoms, and the more physical symptoms a woman experienced the more likely she was to report having postnatal depression, anxiety and PTS symptoms. There was some evidence of a direct association between perineal trauma and PTS symptoms but no evidence of a direct association between perineal trauma and depression or anxiety. Assessment and management of physical symptoms in the postnatal period may play an important role in reducing both physical and psychological postnatal morbidity.

**Supplementary Information:**

The online version contains supplementary material available at 10.1186/s12884-023-05950-6.

## Introduction

Perineal trauma, involving either naturally occurring tears or episiotomy, is common during childbirth. It has been estimated that 85% of women having a vaginal birth will experience perineal trauma, and at least 70% of these will experience suturing of the wound [1]. Reported rates of perineal trauma for primiparous women vary from 35.1–78.3% for second-degree tears and 5.1–8.3% for third and fourth degree tears that involve obstetric anal sphincter injuries (OASIs). The reported rates for multiparous women are more consistent with second-degree tears reported to be 34.8–39.6% and third- and fourth-degree tears 1.8–2.8% [2]. Episiotomy rates have been reported to be around 18.1% in primiparous women and 5.6% in multiparous women [3]. In the United Kingdom (UK), 22% of women having a vaginal birth undergo episiotomy and 3.5% experience a third‐ or fourth‐degree tears [4].

Perineal trauma following childbirth has been associated with a number of physical and psychological complications [5, 6]. Depending on the degree of perineal trauma, it can cause pain [7–9], urinary and faecal incontinence [10], and sexual dysfunction [11, 12]. It may also negatively affect a woman's day-to-day quality of life [13] and maternal relationships with their infant, partner and family [14]. Dunn et al. (2015) found a relationship between second-degree and more severe perineal tears with symptoms of depression at one month postpartum and continuing to three months postpartum [15], however, the study of psychological outcomes in research into perineal trauma is limited [16]. A number of adverse psychological outcomes have been associated with perineal pain, for example, postnatal depression and post-traumatic stress disorder [17, 18] which highlights that it may be the experience of associated pain and other symptoms rather than the perineal trauma that is associated with poor psychological outcomes.

Similarly, it is also unclear from the current literature whether it is perineal trauma or context of the trauma, that impacts on postpartum mental health. For example, Wiseman et al. (2019) found that the quality of care received during the intrapartum and immediate postpartum period had an influence on how women perceived and managed their perineal morbidity [19]. Even after controlling for mode of birth, Molyneux, Fowler and Slade (2021) found women with an OASI reported a more negative overall birth experience compared to those with a 1st/2nd degree tear which had been sutured [20]. Those with an episiotomy reported feeling less involved in decision making processes during their birth.

Considering the large number of women experiencing perineal trauma and its potential impact on postpartum mental health, a better understanding is needed of the relationship between perineal trauma and postpartum psychological outcomes and the factors which may mediate that relationship such as physical symptoms and satisfaction with care. Understanding the underlying mechanisms that may lead to depression or other psychological problems after perineal trauma would help identify who is most vulnerable and how and when to intervene.

## Methods

### Aim

This study aims to determine the associations between perineal trauma and psychological outcomes (depression, anxiety, and PTS symptoms) reported by women three months after giving birth and to explore factors (physical symptoms and experience of care) that could mediate relationships between perineal trauma and maternal psychological outcomes.

### Study design and setting

We conducted a secondary analysis of data from a cross-sectional population-based survey, the National Maternity Survey (NMS) 2014 [21]. The NMS 2014 was a postal survey of maternal and infant health and care conducted in England by the National Perinat al Epidemiology Unit (NPEU). Women were eligible to participate in the survey if they were aged 16 years and over and had a baby in England over a two-week period in January 2014.

### Participants

The sample for the NMS survey was a random sample of eligible women identified from birth registrations by the Office for National Statistics (ONS). Each woman who met the inclusion criteria was allocated a completely random number, once the data were extracted from the processing system. The numbers for the selected sample were then generated by VBA code within Microsoft Access. Mothers of babies who had died in the months following registration of their birth were replaced in the survey sample. Women in the sample were contacted directly by ONS with each woman receiving a package containing a letter inviting them to participate in the survey along with the survey questionnaire, information leaflet, information sheet in 19 languages and a Freephone contact number. The women were asked to respond by either completing and returning the questionnaire to the NPEU by post, or online using a link to the NPEU website which included a unique reference number and individual password, or on the phone with an interviewer at the NPEU and interpreter if necessary. Women who did not have a vaginal birth were excluded from this analysis.

### Exposure, mediators, outcomes, and covariates

The main explanatory variable in this analysis was perineal trauma, defined based on women’s self-reported experience of an episiotomy or tear during birth. It was coded as an ordinal variable with increasing values of the variable representing increasing levels of trauma. Women who reported not having an episiotomy or tear, i.e. those with an intact perineum and no other trauma, were on the lowest level of this variable. On the next level were women without an episiotomy who had a first- or second-degree tear not requiring stitches. Next were women with a first- or second-degree tear requiring stitches, including labial suturing, suturing of the vaginal wall, and perineal body trauma. Women who had an episiotomy were the penultimate level of severity on this scale and were considered to be a higher degree of trauma than those who had first- or second-degree tears without an episiotomy. The level of highest severity were women who had suffered an obstetric anal sphincter injury (OASI).

The psychological outcomes in this analysis were postnatal depression, anxiety and post-traumatic stress (PTS) symptoms. Postnatal depression was measured using the Edinburgh Postnatal Depression Scale (EPDS) which measures experiences in the previous seven days. Women with EPDS scores greater than 12 were classified as having symptoms of postnatal depression. The presence of anxiety was determined from the EPDS-3A subscale; women with scores of six or more were classified as having postnatal anxiety. For PTS symptoms, women were asked if they had experienced hyperarousal (sleep problems not relating to the baby, difficulties concentrating) or intrusion (‘flashbacks’ to the labour or birth) at any time 10 days, one month or three months after the birth; the presence of intrusions/flashbacks and at least one other symptom was considered to be indicative of PTS symptoms.

Physical symptoms and satisfaction with the care received during labour and birth were considered potential mediators of the association between perineal trauma and psychological outcomes. A physical symptoms score was obtained by adding up the number of symptoms that women experienced at any point 10 days, one month or three months after the birth. Symptoms considered were: painful stitches or wound, wound infection, urine incontinence, backache, and painful sex. Satisfaction with care was measured using a 5-point Likert-type response scale rating the level of satisfaction in response to the question: “Overall, how satisfied or dissatisfied were you with the maternity care you received during your care during labour and birth?”. Response levels were: ‘very satisfied’, ‘satisfied’, ‘neither satisfied nor dissatisfied’, ‘dissatisfied’ or ‘very dissatisfied’.

Information on maternal characteristics including age, ethnic group, age left full-time education, parity (defined by whether a woman had previously had a baby), self-identified antenatal anxiety and depression, and infant characteristics including birthweight, gestation, and mode of birth, were used to describe the sample and in subsequent analyses.

### Statistical analysis

The sample of women included in the analysis was described using means and standard deviations for characteristics such as age, birthweight, and gestation, and counts and proportions of categorical characteristics such as ethnic group, education, parity and mode of birth. Counts and proportions of categorical exposure and outcome variables were also tabulated.

**Fig. 1:**
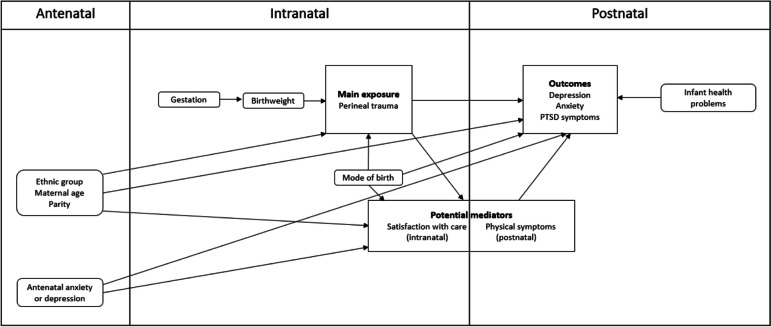
Directed acyclic graph of conceptual relationships between variables in the analysis

A directed acyclic graph was used to map key potential causal relationships between the main exposure, potential mediators, outcomes, and covariates, and to guide subsequent analyses (Fig. 1). The associations between the main exposure and potential mediators or outcomes were explored using ordinary logistic regression for the binary outcome variables including depression, anxiety and PTS symptoms, and using ordinal logistic regression for the ordinal mediator variables including the physical symptoms and satisfaction with care scores. Unadjusted and adjusted estimates of association with 95% confidence intervals and *p*-values were reported. Adjustment for covariates was guided by the nature of potential causal relationships observed in the directed acyclic graph. Potential mediation was assessed based on the association between the exposure and potential mediator, and between the potential mediator and outcome.

To handle missingness in variables where more than 5% of observations were missing, all regression analyses were conducted on multiple imputed datasets by performing sequential imputation of each of the missing variables using chained equations with all other variables in the estimation models used as predictors in the imputation models. The number of imputed datasets was guided by the rule-of-thumb setting it at not less than a hundred times the largest fraction of missing information [22].

All analyses were conducted using Stata SE version 15 (Stata Corporation, College Station, Texas, USA).

### Ethical approval

The National Research Ethics Service (NRES) committee for Yorkshire and The Humber – Humber Bridge provided ethical approval for the NMS 2014 on 28^th^ February 2014 (research ethics committee reference 14/YH/0065).

## Results

### Sample characteristics

Of the 10,000 women invited to participate in the NMS 2014, a total of 4,578 women responded, a response rate of 47%. A total of 1,271 (27.8%) of these women did not have a vaginal birth; further excluding these women resulted in 3,307 women eligible for inclusion in this analysis.

Characteristics of the 3,307 eligible women are summarised in Table 1.

**Table 1:** Characteristics of women included in the dataset

Characteristic	*N* = 3,307
Maternal age in years, mean (SD)	30.5 (5.3)
Ethnic group, n (%)	
White	2,730 (82.6)
Non-white	502 (15.2)
Missing	75 (2.3)
Age when left fulltime education, n (%)	
< 17	569 (17.2)
17 to 18	875 (26.5)
19 or more or still in education	1,817 (54.9)
Missing	46 (1.4)
Parity, n (%)	
Primiparous	1,319 (39.9)
Multiparous	1,607 (48.6)
Missing	381 (11.5)
Birthweight in grams, mean (SD)	3,351.2 (598.4)
Gestation in weeks, mean (SD)	39.6 (1.9)
Mode of birth, n (%)	
Spontaneous vaginal	2,642 (79.9)
Instrumental	665 (20.1)
Antenatal anxiety or depression, n (%)	
No	2,855 (86.3)
Yes	452 (13.7)

They were on average 30.5 years old. Most of the women, 82.6%, were of white ethnicity. More than half of the women were aged 19 or more years when they left full-time education, although about a quarter were aged 17 to 18 years and just under a fifth were aged 16 years or less. Nearly 40% of the women had given birth to their first baby. The mean birthweight of the babies was 3.4kg, with an average of 39.6 weeks of completed gestation. A fifth of women had either ventouse- or forceps-assisted instrumental birth. The prevalence of antenatal anxiety or depression was 13.7%.

### Perineal trauma

A fifth of women had an intact perineum. Just under 10% reported having a tear that did not need stitches while a third had a tear needing stitches. Nearly a quarter of women reported having an episiotomy during the birth, and about 7% were classified as having had an obstetric anal sphincter injury (Table 2).

**Table 2:** Definition and prevalence of perineal trauma

Type of trauma	N (%)
Intact perineum	683 (20.7)
Tear not requiring stitches	324 (9.8)
Tear requiring stitches	1,100 (33.3)
Episiotomy	771 (23.3)
Obstetric anal sphincter injury	227 (6.9)
Unknown (missing or unsure)	202 (6.1)

### Psychological outcomes

The prevalence of postnatal depression was 9.0%. A similar proportion of women, 9.4%, reported having PTS symptoms. A slightly larger proportion of women, 12.2%, had EPDS-3A scores greater than six and were classified as having anxiety (Table 3).

**Table 3:** Prevalence of physical symptoms and psychological outcomes

**Psychological outcomes**	***N* = 3,307**
Depression, n (%)	
No	2,879 (87.1)
Yes	299 (9.0)
Missing	129 (3.9)
Anxiety, n (%)	
No	2,802 (84.7)
Yes	402 (12.2)
Missing	103 (3.1)
PTS symptoms, n (%)	
No	2,995 (90.6)
Yes	312 (9.4)
**Physical symptoms**	***N* = 3,307**
Painful stitches or wound, n (%)	
No	1,823 (55.1)
Yes	1,470 (44.5)
Missing	14 (0.4)
Wound infection, n (%)	
No	3,042 (92.0)
Yes	251 (7.6)
Missing	14 (0.4)
Urine incontinence, n (%)	
No	2,525 (76.4)
Yes	768 (23.2)
Missing	14 (0.4)
Backache, n (%)	
No	1,740 (52.6)
Yes	1,553 (47.0)
Missing	14 (0.4)
Painful sex, n (%)	
No	2,624 (79.4)
Yes	669 (20.2)
Missing	14 (0.4)
Physical symptoms score, n (%)	
0	864 (26.1)
1	992 (30.0)
2	797 (24.1)
3	461 (13.9)
4	153 (4.6)
5	26 (0.8)
Missing	14 (0.4)
**Satisfaction with care during labour and birth**	***N* = 3,307**
Satisfaction score, n (%)	
0 (Very dissatisfied)	74 (2.2)
1 (Dissatisfied)	131 (4.0)
2 (Neutral)	157 (4.8)
3 (Satisfied)	831 (25.1)
4 (Very satisfied)	2,083 (63.0)
Missing	31 (0.9)

All symptoms and outcomes measured at 10 days, 1 month, or 3 months after the birth, except depression and anxiety which are measured using the Edinburgh Postnatal Depression Scale which assesses symptoms occurring in the most recent seven days. *PTS* is post-traumatic stress

### Physical symptoms and satisfaction with care

About a quarter of women had none of the physical symptoms (painful wound, wound infection, urine incontinence, backache or painful sex); 30% had one symptom, and another quarter had two symptoms; the remaining women had three or more physical symptoms (Table 3). The prevalence of all physical symptoms declined over time except for painful sex the prevalence of which rose over time likely coinciding with patterns of resumption of sex after the birth (Supplementary Table 1). Most women reported being very satisfied (63.0%) or satisfied (25.1%) with the care they received during labour and birth; 11% of women felt neutral, dissatisfied or very dissatisfied.

### Association between perineal trauma and potential mediators

There was strong evidence of association between perineal trauma and physical symptoms, with a 47% increase in the proportional odds of a unit increase in the physical symptoms score comparing women in adjacent levels of severity of perineal trauma when adjusting for ethnic group, maternal age, parity and mode of birth (proportional odds ratio (pOR) 1.47, 95%CI 1.38 to 1.57, *p*-value < 0.001) (Table 4; cross-tabulations in Supplementary Table 2). There was no evidence of adjusted association between perineal trauma and satisfaction with care (pOR 0.96, 95%CI 0.90 to 1.03, *p*-value 0.308).

**Table 4:** Association between perineal trauma and potential mediators

**Potential mediator**	**Unadjusted**			**Adjusted**^a^		
	**OR**^b^	**95%CI**	***p*-value**	**OR**^b^	**95%CI**	***p*-value**
Physical symptoms score	1.72	1.63 to 1.82	< 0.001	1.47	1.38 to 1.57	< 0.001
Satisfaction with care	0.93	0.88 to 0.99	0.014	0.96	0.90 to 1.03	0.308

^a^Adjusting for ethnic group, maternal age, parity, and mode of birth

^b^Proportional odds ratio

### Association between potential mediators and postnatal psychological outcomes

There was very strong evidence of association between each potential mediator and each outcome. A unit increase in the number of physical symptoms was associated with 44%, 38%, and 90% increased odds of postnatal depression, anxiety, and PTS symptoms, respectively, adjusting for ethnic group, maternal age, parity, mode of birth, infant health problems, antenatal anxiety or depression, and perineal trauma (Table 5a). A unit increase in the satisfaction with care score was associated with 16%, 2%, and 37% reduced adjusted odds of postnatal depression, anxiety, and PTS symptoms, respectively (Table 5b).

**Table 5:** Association between potential mediators and postnatal psychological outcomes

**Outcome**	**Unadjusted**			Partly adjusted*			Further adjusted**		
	**OR**	**95%CI**	***p*-value**	**OR**	**95%CI**	***p*-value**	**OR**	**95%CI**	***p*-value**
***a: Physical symptoms score***									
Depression	1.47	1.33 to 1.61	< 0.001	1.43	1.28 to 1.60	< 0.001	1.44	1.28 to 1.62	< 0.001
Anxiety	1.41	1.30 to 1.53	< 0.001	1.37	1.25 to 1.51	< 0.001	1.38	1.25 to 1.52	< 0.001
PTS symptoms	2.03	1.83 to 2.24	< 0.001	1.91	1.70 to 2.14	< 0.001	1.90	1.69 to 2.14	< 0.001
***b: Satisfaction with care during labour and birth***									
Depression	0.79	0.70 to 0.88	< 0.001	0.84	0.74 to 0.95	0.004	0.84	0.74 to 0.94	0.004
Anxiety	0.91	0.82 to 1.01	0.088	0.98	0.87 to 1.10	0.735	0.98	0.87 to 1.10	0.694
PTS symptoms	0.67	0.60 to 0.74	< 0.001	0.70	0.63 to 0.78	< 0.001	0.70	0.63 to 0.78	< 0.001

^*^Adjusting for ethnic group, maternal age, parity, mode of birth, infant health problems, and antenatal anxiety or depression, and further** for perineal trauma. *PTS* is post-traumatic stress

### Association between perineal trauma and postnatal psychological outcomes

There was no evidence of association between perineal trauma and depression (OR 1.03, 95%CI 0.90 to 1.17, *p*-value 0.684) or anxiety (OR 1.03, 95%CI 0.92 to 1.15, *p*-value 0.657) after further adjustment for physical symptoms in addition to adjusting for ethnic group, maternal age, parity, mode of birth, infant health problems, antenatal anxiety or depression (Table 6). Similarly, there was no evidence of association between perineal trauma and depression (OR 1.05, 95%CI 0.92 to 1.19, *p*-value 0.493) or anxiety (OR 1.05, 95%CI 0.94 to 1.17, *p*-value 0.436) after further adjustment for satisfaction with care in addition to adjusting for ethnic group, maternal age, parity, mode of birth, infant health problems, antenatal anxiety or depression. There was some evidence of association between perineal trauma and PTS symptoms after further adjusting for satisfaction with care in addition to adjustment for ethnic group, maternal age, parity, mode of birth, infant health problems, and antenatal anxiety or depression (OR 1.19, 95%CI 1.04 to 1.36, *p*-value 0.010) and weak evidence of association after further adjusting for physical symptoms (OR 1.13, 95%CI 0.99 to 1.30, *p*-value 0.067).

**Table 6:** Association between perineal trauma and postnatal psychological outcomes

**Outcome**	**Unadjusted**			Partly adjusted*			Further adjusted** (1)			Further adjusted** (2)		
	**OR**	**95%CI**	***p*-value**	**OR**	**95%CI**	***p*-value**	**OR**	**95%CI**	***p*-value**	**OR**	**95%CI**	***p*-value**
Depression	1.00	0.90 to 1.10	0.967	1.05	0.92 to 1.19	0.503	1.03	0.90 to 1.17	0.684	1.05	0.92 to 1.19	0.493
Anxiety	1.02	0.94 to 1.12	0.589	1.04	0.93 to 1.16	0.474	1.03	0.92 to 1.15	0.657	1.05	0.94 to 1.17	0.436
PTS symptoms	1.30	1.17 to 1.44	< 0.001	1.19	1.05 to 1.36	0.009	1.13	0.99 to 1.30	0.067	1.19	1.04 to 1.36	0.010

^*^Adjusting for ethnic group, maternal age, parity and mode of birth, infant health problems, antenatal anxiety or depression, and further** for (1) physical symptoms or (2) satisfaction with care. *PTS* is post-traumatic stress

## Discussion

This study examined the association between perineal trauma and postnatal psychological outcomes three months postpartum in a sample of women who had a vaginal birth in England in 2014. We further explored the potential role of women’s postnatal experience of physical symptoms and satisfaction with the care received during labour and birth in mediating the association between perineal trauma and postnatal psychological outcomes. There was no evidence of association between severity of perineal trauma and depression or anxiety, however women who experienced perineal trauma were more likely to experience physical symptoms, and the more physical symptoms a woman experienced the more likely she was to report having postnatal depression, anxiety, and PTS symptoms. We found no evidence of association between the experience of perineal trauma and satisfaction with care, although women who were satisfied with the postnatal care they received were less likely to report adverse postnatal mental and physical outcomes; thus, there was no evidence of a mediating role of satisfaction with care in the association between perineal trauma and the outcomes.

The lack of association between perineal trauma and depression reflects those of Crockall et al. (2018) In a mixed methods review Crockall et al. (2018) found that while perineal trauma can have a negative impact on psychological well-being, the literature is conflicted and the findings mixed for depression [23]. Jahani Shoohab et al. (2019), in a qualitative study, found that women were able to recover their psychological wellbeing following perineal trauma with the support of their family and community. Also in keeping with our findings, improved physical function had a key role in regaining emotional well-being and enjoying daily life [18]. Our findings suggest that psychological screening for women who have experienced perineal trauma, a poor birth experience or subsequent physical symptoms is needed.

The lack of association between perineal trauma and psychological outcomes and the consistent relationship of physical symptoms with all psychological outcomes suggests that the experience of physical symptoms in the postpartum period could mediate the association between perineal trauma and adverse postnatal psychological outcomes. The most commonly reported physical symptoms in the first three months in this study were backache and painful stitches which is similar to the findings of Woolhouse et al. (2014) [24]. A unit increase in the number of physical symptoms was associated with significantly increased odds of postnatal depression, anxiety, and PTS symptoms even after adjusting for other variables. Again, this is in keeping with Woolhouse et al. (2014) who found the odds of reporting concurrent depressive symptoms at three months postpartum were increased almost four-fold for women reporting 3–4 health problems when compared to women reporting 0–2 health problems, and more than six-fold for women reporting five or more health problems [24].

While evidence of a dose response relationship suggests that physical health problems may contribute to psychological problems, the relationship between maternal mental health and physical health is likely to be bi-directional. Physical symptoms may create a significant psychological burden and mental health problems may exacerbate or delay recovery from physical health symptoms by factors such as delayed help-seeking and poor engagement with treatment [24, 25].

Our findings showed no relationship between perineal trauma and satisfaction with care which differs from Molyneux, Fowler and Slade (2021) who found women with an OASI reported a significantly more negative birth experience [20]. However, Molyneux et al. (2021) collected data immediately after birth as opposed to three months after birth. A number of studies have suggested that the timing of the assessment of women's experience of childbirth matters and women may need a longer period of time to work through their experience [20]. Some suggest that many aspects of women’s memories of childbirth experience improve with time [26–28], others [29] found women’s memories became more negative from two months to one year after birth.

### Strengths and limitations

The main strength of the study is the use of a large population-based sample. The survey explored both physical and psychological outcomes and therefore we were able to explore the complex relationships between physical and psychological health. The number of women in the sample who reported an intact perineum or a tear that required no stitches was similar to previously reported prevalence [1]. Episiotomy rates were also similar to those reported in the UK although the percentage of OASIs was higher than expected for a mixed sample of primiparous and multiparous women. The reason for this is unclear; it may be that women are more likely to take part in the survey if they have had a negative or traumatic birth experience or women may be unclear about the degree of trauma they experienced.

There were a number of potential methodological limitations. The response rate was 47% and survey non-responders were more likely to be younger, living in more deprived areas or born outside the UK. We do not have psychological outcome data for non-responders so cannot comment if women with mental health problems were less likely to respond. The survey was conducted at 3 months postpartum with data about labour and birth being reported retrospectively. While variables such as mode of birth and episiotomy are likely to be accurately reported, more subjective variables such as satisfaction with care during labour and birth may be biased by conditions at the time of reporting. However, completing the survey at 3 months postpartum minimises socially desirable responses by completing the questionnaire independently from the care provider and avoids the initial emotional responses of the early postpartum period. A further limitation is that some of the mediators and outcomes relied on single items rather than validated, standardised measures. In addition, the data were collected in 2014 and a number of changes may have been introduced into practice that could impact on the prevalence of perineal trauma and its association with physical symptoms three months after birth, for example, the recent care bundle initiative to prevent obstetric anal sphincter injury (OASI-CB) [30, 31].

### Implications for policy and practice

Concerns have been expressed about a lack of knowledge, in both health professionals and women, of how to identify and manage perineal trauma [32]. These concerns and the rise in OASI rates in the UK, led to a collaboration by the Royal College of Obstetricians and Gynaecologists and the Royal College of Midwives to develop an evidence-based care bundle (OASI-CB) to improve perineal outcomes [30]. The elements of the OASI bundle included: informing women about OASI and identifying what can be done to minimise her risk, recommending an episiotomy when indicated, using the hands to support the perineum at the time of birth, and a thorough rectal examination after birth to detect tears. The care bundle also recommends that these elements should be underpinned by good communication with the woman before, during and after birth. The care bundle was implemented in 16 maternity units in the UK and an evaluation of its implementation using a multicentre, stepped wedge cluster design found that the OASI-CB reduced overall OASI rates but that the rate was unchanged among births assisted by forceps [30, 31]. Similar care bundles have been implemented in other countries [33, 34].

Consideration also needs to be given to longer term care of perineal trauma. Reporting on 11 years outcome data, Wan et al. (2020) describes the benefits of a one stop perineal wound clinic run by a urogynaecologist with trained specialist midwives in perineal care. In addition to women who had OASIs, postpartum women who suffered bowel, bladder, prolapse and sexual problems attended. While the outcomes described by Wan et al. (2020) were all physical symptoms, a one stop multidisciplinary clinic provides opportunities to assess and manage the interplay between physical and mental health. Promoting models of care that integrate physical and psychological assessment has been identified by the World Health Organisation as a priority area for action [25].

Our findings suggest it is important that postpartum women with perineal complications not related to OASIs should also have an opportunity for similar assessment and support. This is supported by Williams et al. (2007) who found enduring postnatal perineal morbidity is common in women with intact perineum after childbirth as well as all grades of perineal trauma and perineal pain maybe experienced without perineal trauma or with lower degree tears [35]. The assessment and management of pain and other postpartum symptoms for all women, not just degree of perineal trauma, is key. A recent review by NICE for postnatal care guidance [36] highlighted that in practice it is often deemed acceptable to normalise perineal pain in contrast to the pain experienced following other clinical events. The current findings would suggest that encouraging healthcare professionals to offer, or women themselves to ask for, early assessment and appropriate management for pain and other physical symptoms would be beneficial for longer term physical and mental health outcomes.

## Conclusion

We found evidence of association between perineal trauma and physical symptoms: women who experienced perineal trauma were more likely to experience physical symptoms, and that the more physical symptoms a woman experienced the more likely she was to report having postnatal depression, anxiety, and PTS symptoms. These findings suggest that the experience of physical symptoms in the postpartum period could mediate the association between perineal trauma and adverse postnatal psychological outcomes. Assessment and management of physical postnatal symptoms may play an important role in reducing psychological as well as physical postnatal morbidity for new mothers.

### Supplementary Information

**Additional file 1: Table S1.** Prevalence of physical symptoms and psychosocial outcomes over time. **Table S2.** Distributions of mediator and outcome variables across levels perineal trauma.

## Data Availability

The datasets used and/or analysed during the current study are available from the corresponding author on reasonable request.
