# Supplementary material for: The relationship between perineal trauma and postpartum psychological outcomes: a secondary analysis of a population-based survey
Source: BMC Pregnancy Childbirth. 2023 Sep 6;23:639. doi: 10.1186/s12884-023-05950-6 (PMC10481495; doi:10.1186/s12884-023-05950-6)
Supplement: Supplementary file 1 — Additional file 1: Table S1. Prevalence of physical symptoms and psychosocial outcomes over time. Table S2. Distributions of mediator and outcome variables across levels perineal trauma. [file 12884_2023_5950_MOESM1_ESM.docx]

**Supplementary tables**

**Table S1: Prevalence of physical symptoms and psychosocial outcomes over time**

| **Symptoms and outcomes** | **Prevalence* in the postnatal period** | | | |
| --- | --- | --- | --- | --- |
|  | **10 days** | **1 month** | **3 months** | **Any time** |
| Sleep problems | 5.5% | 4.9% | 4.0% | 10.6% |
| Flashbacks | 11.7% | 6.0% | 3.6% | 14.3% |
| Difficulties concentrating | 15.6% | 13.1% | 8.0% | 22.3% |
| Painful stitches or wound | 40.0% | 13.8% | 2.9% | 44.6% |
| Wound infection | 6.1% | 2.5% | 0.6% | 7.2% |
| Urine incontinence | 16.9% | 11.7% | 6.8% | 23.3% |
| Backache | 30.3% | 23.5% | 20.1% | 47.2% |
| Painful sex | 3.6% | 11.1% | 10.7% | 20.3% |
| * Responses from 3,293 women | | | | |

**Table S2: Distributions of mediator and outcome variables across levels perineal trauma**

| **Mediator/outcome** | **Perineal trauma** | | | | | |
| --- | --- | --- | --- | --- | --- | --- |
|  | Intact perineum | Tear not requiring stitches | Tear requiring stitches | Episiotomy | Obstetric anal sphincter injury | Unknown (missing or unsure) |
| Satisfaction with care |  |  |  |  |  |  |
| 0 (Very dissatisfied) | 18 (2.6%) | 8 (2.5%) | 13 (1.2%) | 24 (3.1%) | 9 (4.0%) | 2 (1.0%) |
| 1 (Dissatisfied) | 29 (4.3%) | 14 (4.3%) | 30 (2.7%) | 39 (5.1%) | 14 (6.2%) | 5 (2.5%) |
| 2 (Neutral) | 35 (5.1%) | 18 (5.6%) | 44 (4.0%) | 41 (5.3%) | 10 (4.4%) | 9 (4.5%) |
| 3 (Satisfied) | 153 (22.4%) | 83 (25.6%) | 260 (23.6%) | 207 (26.9%) | 67 (29.5%) | 61 (30.2%) |
| 4 (Very satisfied) | 443 (64.9%) | 200 (61.7%) | 747 (67.9%) | 453 (58.8%) | 124 (54.6%) | 116 (57.4%) |
| Missing | 5 (0.7%) | 1 (0.3%) | 6 (0.6%) | 7 (0.9%) | 3 (1.3%) | 9 (4.5%) |
|  |  |  |  |  |  |  |
| Physical symptoms score |  |  |  |  |  |  |
| 0 | 293 (42.9%) | 118 (36.4%) | 263 (23.9%) | 96 (12.5%) | 26 (11.5 %) | 68 (33.7%) |
| 1 | 261 (38.2%) | 122 (37.7%) | 324 (29.5%) | 175 (22.7%) | 56 (24.7%) | 54 (26.7%) |
| 2 | 90 (13.2%) | 54 (16.7%) | 294 (26.7%) | 243 (31.5%) | 69 (30.4%) | 47 (23.3%) |
| 3 | 33 (4.8%) | 25 (7.7%) | 167 (15.2%) | 171 (22.2%) | 43 (18.9%) | 22 (10.9%) |
| 4 | 4 (0.6%) | 5 (1.5%) | 41 (3.7%) | 69 (9.0%) | 27 (11.9%) | 7 (3.5%) |
| 5 | 1 (0.2%) | 0 (0.0%) | 8 (0.7%) | 13 (1.7%) | 3 (1.3%) | 1 (0.5%) |
| Missing | 1 (0.2%) | 0 (0.0%) | 3 (0.3%) | 4 (0.5%) | 3 (1.3%) | 3 (1.5%) |
|  |  |  |  |  |  |  |
| Depression |  |  |  |  |  |  |
| No | 595 (87.1%) | 279 (86.1%) | 983 (89.4%) | 675 (87.6%) | 196 (86.3%) | 151 (74.6%) |
| Yes | 62 (9.1%) | 34 (10.5%) | 86 (7.8%) | 70 (9.1%) | 22 (9.7%) | 25 (12.4%) |
| Missing | 26 (3.8%) | 11 (3.4%) | 31 (2.8%) | 26 (3.4%) | 9 (4.0%) | 26 (12.9%) |
|  |  |  |  |  |  |  |
| Anxiety |  |  |  |  |  |  |
| No | 577 (84.5%) | 277 (85.5%) | 954 (86.7%) | 647 (83.9%) | 193 (85.0%) | 154 (76.2%) |
| Yes | 84 (12.3%) | 37 (11.4%) | 125 (11.4%) | 103 (13.4%) | 29 (12.8%) | 24 (11.9%) |
| Missing | 22 (3.2%) | 10 (3.1%) | 21 (1.9%) | 21(2.7%) | 5 (2.2%) | 24 (11.9%) |
|  |  |  |  |  |  |  |
| PTS symptoms |  |  |  |  |  |  |
| No | 639 (93.6%) | 301 (92.9%) | 1,008 (91.6%) | 670 (86.9%) | 195 (85.9%) | 182 (90.1%) |
| Yes | 44 (6.4%) | 23 (7.1%) | 92 (8.4%) | 101 (13.1%) | 32 (14.1%) | 20 (9.9%) |
